# Supporting women’s research in predominantly undergraduate institutions: Experiences with a National Science Foundation ADVANCE Institutional Transformation Award

**DOI:** 10.3389/fpsyg.2022.817269

**Published:** 2022-09-16

**Authors:** Vita C. Rabinowitz, Virginia Valian

**Affiliations:** Hunter College and the Graduate Center, The City University of New York, New York, NY, United States

**Keywords:** ADVANCE, research productivity, accumulation of disadvantage, sustainability of equity efforts, faculty development, teaching-intensive institutions, gender equity, women faculty

## Abstract

This paper describes the Gender Equity Project (GEP) at Hunter College of the City University of New York (CUNY), funded by the U. S. NSF ADVANCE Institutional Transformation Award (ITA) program. ADVANCE supports system-level strategies to promote gender equity in the social and natural sciences, but has supported very few teaching-intensive institutions. Hunter College is a teaching-intensive institution in which research productivity among faculty is highly valued and counts toward tenure and promotion. We created the GEP to address the particular challenges that faculty, especially White women and faculty of color, face in maintaining research programs and advancing in their careers at teaching-intensive institutions. During the course of the ADVANCE award, its centerpiece was the Sponsorship Program, a multifaceted paid mentorship/sponsorship program that paired each participant with a successful scholar in her discipline. It offered extensive professional development opportunities, including interactive workshops and internal grants to support research. The GEP helped change key policies and practices by ensuring that all faculty were treated fairly in areas like provision of research start-up funds and access to guidance on how to prepare for tenure and promotion. Qualitative and quantitative evidence suggests that participation in the Sponsorship Program boosted research productivity and advanced the careers of many of the women who participated; the Program was highly rated by all participants. Some of the policy and practice changes that the GEP helped bring about were sustained at Hunter beyond the award period and some were adopted and disseminated by the central office of CUNY. However, we were not able to sustain the relatively expensive (but cost-effective) Sponsorship Program. We share the lessons we learned, including that creating a diverse, successful social and natural scientific workforce requires sustained support of female faculty employed at teaching-intensive colleges. We acknowledge the difficulties of sustaining gains, and offer ideas about how to make the case for gender equity when women seem to be doing “well enough.” We underscore the imperative of building support for women’s research in teaching-intensive institutions, where most women scientists are employed, and well over 90% of all college students—a disproportionate percentage of whom are female, minoritized, or both—are educated.

## Introduction

Our goals in this paper are to:

• Argue for the importance of remedying the particular challenges that faculty, particularly White women and faculty who are Black, Indigenous, and People of Color (BIPoC), face at teaching-intensive institutions.
• Describe the creation and delivery of a comprehensive faculty development program aimed at supporting women’s research careers at a teaching-intensive institution that also requires research productivity.
• Detail the changes the NSF ADVANCE-funded Gender Equity Project (GEP) led and inspired at the City University of New York (CUNY).
• Share the lessons we learned, paramount among them that creating a diverse, successful social and natural scientific workforce requires *sustained* attention to and support of female faculty employed at predominantly undergraduate, teaching-intensive colleges.
• Review the implications of our analyses and recommend steps forward.

## Female faculty in primarily undergraduate institutions: underfunded, overlooked, and disadvantaged

### Who has received NSF ADVANCE Institutional Transformation Awards, and why?

The NSF ADVANCE program is the largest, most comprehensive and most prestigious program to promote gender equity in U. S. academic science and engineering. Since 2001, the NSF ADVANCE program has invested more than $270M, most of it *via* its Institutional Transformation Awards (ITA), to increase the representation and advancement of women scientists through systemic change in institutions. Of the 70 universities that have thus far received ITAs, only five are outside the classification of very high (R1) or high (R2) research activity. The first cohort, funded in 2001/2002, included two such schools – Hunter College and the University of Puerto Rico at Humacao. Over the subsequent 20 years, only three more schools outside the research-intensive framework joined the ADVANCE IT awardees. Thus, the schools that could benefit the most have received the fewest awards. (Other award mechanisms, such as Partnership and Adaptation awards, and, earlier, PAID awards, are more evenly distributed. Those awards provide much less money than IT awards.)

There are several possible reasons why so few such schools have received ITAs, and why most of the published literature on the advancement of women scientists has been conducted by researchers at research-intensive universities. For one thing, what happens at prestigious universities attracts more attention than what happens at other institutions simply because they are seen as better and more important. For another, faculty gender imbalances in representation—among other gender inequities in salary, research space, academic rank—have historically been larger at research-intensive universities than they are in teaching-intensive institutions (Bradburn and Sikora, 2002), though the smaller gender disparities in salary in teaching-intensive institutions is likely due to salary compression. For yet another, teaching and service obligations of faculty in teaching-intensive institutions are so high—and institutional infrastructure support for research and writing is so low—that faculty at such institutions lack the time and resources to prepare competitive applications. Finally, one criterion that NSF and other federal agencies use to make awards is “institutional environment,” a criterion that consistently works against predominantly undergraduate institutions.

Extensive research on gender schemas and our own experience in predominantly undergraduate institutions suggests additional, less explicit, reasons for the disparity in ITA awards. Faculty at institutions like Hunter are less likely than faculty at research-intensive institutions to be part of professional networks that supply information about how to be successful in the domains of grant-writing, research, and publication. Even if faculty apply for funding and have excellent ideas, they are not necessarily able to organize and write those ideas in grant-appropriate prose, nor will they know to incorporate grant-winning strategies. As one example, Valian recalls going red with embarrassment at a chance meeting in 2001 with a researcher who was also applying for an ADVANCE ITA. That researcher mentioned that their PIs included deans and the provost. Valian immediately saw the obvious importance of including upper-level administrators, but she had had too little experience with institutional proposals to have had such strategies in mind. Nor was she part of a professional network that would have supplied her with relevant information.

*To sum up*, there are many reasons so few teaching-intensive institutions have received ITA awards—starting with the lack of time and grant-development resources necessary to craft competitive applications. Also among those reasons are the hidden disadvantages of being outside the prestigious institutions that confer professional legitimacy and offer formal and informal networks that provide insider knowledge.

### Neglected: Female faculty in predominantly undergraduate institutions

The challenges and disadvantages that women experience in academia are well-documented, in several cases by researchers funded by the NSF ADVANCE program (e.g., Holman et al., 2018; O’Meara et al., 2018; Stewart and Valian, 2018; Lundberg and Stearns, 2019; Casad et al., 2021). Women in leadership face even more challenges (e.g., Lyness and Grotto, 2018). We focus here on what we think is a neglected group: female researchers in predominantly undergraduate institutions.

Teaching-intensive institutions vary greatly in the extent to which faculty are expected to conduct research. Generally, research activity is greater in four- than in two-year institutions, and in master’s degree-granting institutions than in baccalaureate-granting institutions. But even in many community colleges [including all seven of those in the City University of New York (CUNY)], faculty are tenured and promoted based in part on their research productivity.

Attention to researchers at such institutions^1^ —master’s and baccalaureate-granting colleges, regional colleges, and community colleges—is important for two reasons. First, those institutions, which are far more numerous than elite research institutions, are the places where most women and Black, Indigenous, and People of Color (BIPoC) faculty are employed and conduct research. Second, they are the places where the overwhelming majority of students of color, poor students, and immigrant and first-generation college students are educated (Fry and Cilluffo, 2019).

At community colleges across the nation, 15% of students come from the bottom income quintile and only 0.5% come from the top percentile. In contrast, at the Ivy-Plus colleges (Ivy League schools plus Duke, MIT, Stanford, and the University of Chicago), only 4% of students come from the bottom income quintile, while 15% come from the top percentile (Chetty et al., 2020). It is not just Ivy-Plus colleges that fail in their attention to diversity. An analysis of 101 selective publicly funded institutions shows how little improvement there has been in enrollment of Black and Latinx students since 2000, and how many institutions fail to enroll Black and Latinx students at rates comparable to their presence in their state population (Nichols, 2020). A disturbing 75% of those schools received failing grades for enrollment of Black students, while only 9% received an A; 50% received failing grades for enrollment of Latinx students, while 14% received an A (Nichols, 2020).

Students who come to college with few advantages profit disproportionately from experiential learning, and particularly from authentic, sustained opportunities like undergraduate research (Collins et al., 2017; Stellar, 2017). Exposure to a diverse, research-active faculty and authentic, substantial research experience is critical in showing students that they can create as well as consume knowledge and that productive, successful people in academia are not all White men (Thiry et al., 2012; Lopato, 2017; Fox Tree and Vaid, 2022).

As things stand, however, instead of equalizing opportunity, academia in the United States perpetuates inequality. A staggering 22% of United States faculty have a parent with a PhD (Morgan et al., 2022). For people who earned PhDs and did not go on to become faculty, 11% had a parent with a PhD. For people born at similar times as faculty, less than 1% have a parent with a PhD. Faculty with a PhD parent also received more support, not just financial support, for their ambitions. One way to change those numbers, we suggest, is to support faculty research opportunities at teaching-intensive institutions.

In conclusion, women and people of color are disproportionately represented at teaching-intensive institutions, both as faculty and as students. The failure to support such scientists wastes the human capital of the faculty and compromises the future of the potential next generation of scientists, especially female students and those from underserved groups. Support for women and BIPoC faculty is thus important for increasing and democratizing the nation’s research pool.

### What is life like for faculty at a teaching-intensive institution?

CUNY, of which Hunter is a part, is one of the very few institutions across the nation that are reliable engines of social mobility, meaning that they propel students from the lowest rungs of the economic latter to the middle class and beyond. Along with CUNY are some undergraduate institutions within the California State University and the University of Texas systems (Chetty et al., 2020).

Hunter College, which offers bachelor’s, master’s and professional doctoral degrees in some areas, is an example of a common but particular kind of teaching-intensive institution in which research is highly valued and research productivity is required for tenure and promotion.^2^

In 2002, when Hunter’s ADVANCE IT award began, faculty at Hunter had high teaching (6 courses per year), service, and advising responsibilities. Faculty then and now primarily taught and teach introductory-level, lower-division undergraduate courses, making it difficult to keep up with new developments in their fields (Pannapacker, 2021). In 2001, Hunter offered low-to-no start-up packages to support faculty research and poor support for sabbaticals (50% of salary, which was subsequently increased to 80%). Research facilities were substandard. There was little funding for research-related travel, research assistants, or professional activity. Faculty faced and still face murky expectations about how much research to conduct, and many have little or no access to graduate students, research collaborators, or an intellectual community. Those conditions are a recipe for creating scholars who are disconnected, isolated, and unable to contribute to their disciplines. With each passing year, as disadvantage accumulates, more faculty fall further behind their peers at research-intensive institutions, making it harder for them to compete for grants or develop promising research programs. Faculty development was scarce. Faculty were (and still are) rarely nominated for honors, awards, or opportunities within or beyond the institution.

### The accumulation of disadvantage for women in teaching-intensive institutions

Most college faculty in the United States hold doctoral degrees from R1 or R2 institutions and are socialized early into beliefs and values about the roles of research productivity and excellence in academic careers. College faculty across both research- and teaching- intensive institutions and academic disciplines hold consistent views of the professional hierarchy throughout their careers (Gonzales and Terosky, 2016). Professional legitimacy is associated with having a high academic rank in a highly rated academic program in a prestigious, research-intensive university (O’Meara et al., 2018).

The accumulation of advantage and its corollary, disadvantage, is documented by a study of the effects of institution type on research productivity (Way et al., 2019). Faculty with degrees from equivalently prestigious institutions, and with equivalent productivity before being hired, fare differently depending on the prestige of the institution where they are hired: the people at more prestigious institutions publish an average of five more papers in their first five years of employment than do the people hired at less prestigious institutions. Environments create differential productivity, independent of the relevant attributes of the faculty. And a higher percentage of women than men work in non-research-intensive environments (Stewart and Valian, 2018).

Beyond the disadvantages of working in environments that do not support research, women faculty, wherever they work, accumulate more professional disadvantage than men because they are women. They experience higher levels of sexual and gender harassment (Fitzgerald et al., 1988; MacDonald, 2011), they have less access to mentoring and insider information (King et al., 2012; Lundberg and Stearns, 2019), they receive less positive evaluations throughout their academic training and professional lives (Lundberg and Stearns, 2019; Oleschuk, 2020), they have greater service responsibilities (Guarino and Borden, 2017), and if they are in heterosexual relationships they likely have more family and household responsibilities (Bianchi et al., 2012). Women, especially women of color, and men of color, tend to use different research methods and work on different research topics than White men do; White men’s methods and research areas are more highly valued (Settles et al., 2021). Over time, then, women in teaching-intensive institutions accumulate more and more disadvantages.

### Hidden differences in treatment: The competitive disadvantage for female faculty

Women – White, Black, Asian, and Latina – have joined the full-time tenure-track faculty in greater numbers across all institution types over the past 10 years, but without reaping the same rewards as men, particularly White men. It is relatively straightforward, if not easy, to change obvious inequities. Although institutions do not necessarily monitor salary, for example, how to do so is not complicated, nor is how to remedy gender-linked salary disparities. What remains stubbornly difficult to change are the subtle, often hidden, differences in treatment of men and women that in turn affect how men and women perform and how they are evaluated (Valian, 1998; Stewart and Valian, 2018).

Citations are one example of hidden differences in treatment. Citation counts of publications are increasingly used by tenure and promotion committees throughout higher education to evaluate whether a person has earned advancement and to decide whether to hire someone currently at another institution. Citation counts are sometimes used alone and sometimes as part of the *h* index, an index of how many papers one has published that have been cited that number of times. (An *h* of 35 means that one has published 35 papers, all of which have been cited at least 35 times.) Citations are an objective measure.

What underlying processes do citations reflect? Among others, citations reflect prestige and status factors within academia, with the result that men are unintentionally advantaged and women are unintentionally disadvantaged. Men as first or last author continue to be cited more often and women less often than would be expected (Chatterjee and Werner, 2021, academic medicine; Dworkin et al., 2020, neuroscience). Similarly, Black researchers are cited less than would be expected, especially by their White peers (Bertolero et al., 2020). Another form of citations – reading lists for graduate level courses – benefits men more than women (Skitka et al., 2021, social psychology). Citations are also more common for papers that describe their findings with generic terms – terms that suggest an enduring finding that extends beyond the particular paper – and men are more likely than women to include generics (DeJesus et al., 2021).

Objective measures seem fair, even though they are affected by gender schemas that portray men as more competent than women and as more deserving of credit (Valian, 1998). The finding that professional society awards for researchers in neuroscience go disproportionately to men - except when *h* is taken into account (Melnikoff and Valian, 2019) - can thus be understood as the result of a train of subtle events largely hidden from view. The field is now developing more ways of measuring how gender affects apparently fair metrics that influence the course of a scholar’s career, with advantage (or disadvantage) accumulating over time. Twenty years ago, attention was more narrowly focused on hiring, retention, and promotion (Martell et al., 1996; Valian, 1998). Those remain important, but even as schools make progress on overt problems, the hidden problems remain.

## Sponsorship Program: Rationale, structure, and methods

### Rationale

In our ADVANCE proposal, we hypothesized that, even in an enlightened teaching-intensive institution like Hunter, hidden gender disparities disadvantaged women. In 2001, Hunter had an almost equal number of male and female faculty, and, as our later analyses documented, men and women in the natural and social sciences had equal salaries (in part due to salary compression), equal laboratory and office space, and even more female than male distinguished professors. Hunter seemed to be a post-equity institution where visible problems like unequal representation of men and women at higher ranks had disappeared. Our informal observations suggested that, even so, women had less successful academic careers, and, more subtly, had less influence than men in their departments and were less embedded in professional networks.

The key elements of the Gender Equity Project (GEP), including our signature Sponsorship Program, were designed to address the hidden and not-so-hidden disadvantages that we thought stood in women’s way at Hunter and CUNY. We set out to increase women’s scholarly productivity. Despite their sizable numbers and academic achievements compared to many women at other CUNY campuses, we believed that Hunter’s female faculty in the social and natural sciences lagged behind their male peers in research productivity, career advancement, and satisfaction with support for their research. One goal of the GEP was and remains to advance the research productivity and professional careers of women faculty in the natural and social sciences.

We relied on social science research to establish the principles, policies, and practices of the GEP in general and the Sponsorship Program in particular. The content of our workshops, including assigned readings and exercises, was based in social science. We have described the Sponsorship Program elsewhere (Rabinowitz and Valian, 2007) and summarize it in Table 1, adding new conclusions that reflect what we have learned.

**TABLE 1:** Key elements of the Sponsorship Program.

● The program was open to full-time, tenured or tenure-track female faculty below the rank of full professor from 11 participating social and natural and physical science departments. (We did accept one full professor who was working on a book in a new area.)
● The program featured a rigorous application process that committed the applicant to a set of goals and actions.
● Applicants had to obtain the written approval of the department chair for course release; that release was paid for by the program.
● The program offered internal grants to associates for up to $15,000 per year for research, $5,000 of which went to their sponsors and some of which could be used to purchase course release (with the department chair’s permission).
● Participants could apply twice for an additional year of support, with up to 3 years possible.
● Each program participant was paired with a successful senior scholar, approached personally by one of the GEP co-directors, in the scholar’s discipline or topic area.
● The sponsor had to be outside the participant’s department (and, where possible, outside the college) so as to avoid potential conflicts of interest.
● Sponsors committed to having regular contact with participants, providing written feedback on work products, giving general professional advice and support, and meeting at least once a semester with a GEP co-director to discuss the participant’s progress. In the course of developing the program, we changed from offering sponsors $5,000 per year to $2,500 per semester. That allowed participants to change sponsors if that would be beneficial.
● Mandatory monthly workshops, led by us, by experts within Hunter, by experts within CUNY, and by outside experts, covered such topics as how to negotiate for needed resources, how to present one’s work orally in different formats, how to make the most of summer breaks to advance one’s research, and how to tackle procrastination and other work problems.
● The three GEP co-directors (Valian, Rabinowitz, Dr. Annemarie Nicols-Grinenko, Director of Research and Project Director) actively engaged with all participants, serving as informal mentors and sponsors, supporting them through challenges, intervening when appropriate, and reviewing progress regularly.
● The GEP meetings and social gatherings took place in a convenient, attractive, dedicated space that was removed from departmental and administrative offices.

### Structure

The Sponsorship Program was the centerpiece of the GEP at Hunter College. It was open by application to female faculty below the rank of full professor in the social and natural science departments. We operationally defined “science” as any field that NSF funded. That included Anthropology, Economics, Political Science, Psychology, and Sociology, as well as Biology, Chemistry, Computer Science, Geography (which included geophysics), Mathematics and Statistics, and Physics.

For women at a teaching-intensive institution, funding and release time were necessary to give women resources and time; the program provided $10K/year, out of which participants could fund a course release with their chair’s permission. The program also addressed our perception that men as a whole received more informal information and feedback about how to be professionally successful than women did, and were embedded in more useful professional networks. From the social science literature and our own experiences, we saw professional success as a product of three things: information; the development and deployment of skills and strategies; psychological support. We reasoned that multicomponent interventions were more likely to have an effect than single component interventions. We hypothesized that women and men have both different kinds and numbers of opportunities to receive feedback and different content in the feedback they do receive.

Recent work on “developmental” feedback for aspiring leaders may be relevant to success in academia. Research indicates that men receive more feedback related to how they can become leaders and more challenging and constructive analyses of their performance than women do (King et al., 2012). Comments to women often focus on their interpersonal behavior rather than on how well they perform tasks. An analysis of messages to aspiring political leaders found that women received more empty rah-rah messages while men received more substantive leadership feedback (Doldor et al., 2019).

It does not help women or faculty of color to have a mentor who has low expectations of them or focuses on their interpersonal skills. Interpersonal skills are important, but they are only one component of success in organizations. By having a single mentor, especially an untrained mentor, women run the risk of receiving information and feedback that is not genuinely helpful.

Based on the literature then just developing but now extensive (e.g., McCauley and Martineau, 1998; Packard, 1999, 2003; Blickle et al., 2009; Katz et al., 2009; Allen and Eby, 2011), we rejected the classical mentorship model in favor of a circle of advisors model. The circle of advisors model is similar to the idea of a composite mentor or mentor mosaic or mentor network in which participants receive information and help from a number of sources whom they designate after having analyzed places where they need information and helpful feedback. The classical mentorship model pairs a protégé with a single mentor and assumes that people will grow out of the need for a mentor. Our approach suggests that people need information, feedback, and help throughout their career, though the content changes over time. We thus worked with the faculty associates in the program to help them develop a circle of advisors.

We recognized that faculty also needed intensive attention that they were unlikely to obtain in the normal course of their activities. We thus paired each participant with a senior successful scholar who was paid to provide mentorship and sponsorship—general career guidance and support as well as specific, written feedback on articles and grant proposals. The “sponsor” was paid $5K per year and agreed to a set of activities. Sponsors could not be a member of the faculty member’s department. As part of their application to be in the program, associates indicated what type of person they thought could offer them what they most needed, and were invited to recommend a specific person if they had one in mind.

Finally, the program offered extensive professional development opportunities *via* workshops and support that was otherwise not readily available in departments, in the college, or in the University. The monthly workshops provided information, skills, and supports. Our roster of workshops was initially formed by consulting publications like *The Compleat Academic: A Career Guide* (Zanna and Darley, 1987; Darley et al., 2004) and talking with experts on women’s advancement and colleagues from CUNY. Workshops changed over time as we gained experience with what our associates needed most and what social science had to offer by way of improving institutional and individual effectiveness. Following our analysis of what was holding women back at Hunter College, our focus in the workshops was on hidden—practically invisible, rarely discussed, underappreciated—but ubiquitous aspects of academic life: topics like how to handle rejection; how to start a presentation to draw people in; how to negotiate effectively with a chair for teaching releases, lab space, and other matters; how to use the summers to maximize productivity; how to say no without alienating people; how to make the most out of working with undergraduates, and so on.

Table 2 includes a list of the most commonly offered workshops.

**TABLE 2:** Gender Equity Project (GEP) faculty workshops.

**Mentoring**
● Building and maintaining a circle of advisors
● Advising, mentoring, and sponsoring colleagues
● Mentoring students, staff, and assistants
**Balancing**
● Balancing work responsibilities: Research, teaching, and service
● Balancing work and a personal life
**Writing and publishing**
● Time management and procrastination
● Maximizing research and writing during the summer
● Grant writing
● Successfully handling rejection of papers and grants
**Professional development**
● Curricula vitae (CVs) and cover letters
● Teaching effectively and efficiently
● Attending conferences, public speaking, and presentations
● Tenure and promotion
● Prizes, awards, and other status indicators
● Leadership
**Self-presentation**
● Entitlement and negotiation
● Dealing with conflict
● Social media: Creating a webpage and translating research for the media
● Social and professional networking

### Selection process and participants

Over the 6-year course of the ITA, 30 members of eight academic departments in two divisions of the School of Arts and Sciences participated in the Sponsorship Program as *associates*, or direct beneficiaries of program elements, several for more than one year. The women in the program varied in ethnicity, age, rank, years at Hunter, type of work (laboratory-based and field research; qualitative and quantitative research), and work products (books, peer-reviewed journal articles, grant proposals, talks). Sixty percent were women of color.

Two thirds of all associates were social scientists. (That was unplanned, and may have partly been due to the fact that the principal investigators were social scientists.) In three departments (Geography, Physics, Psychology) most or all of the eligible women joined the program. In two others (Biology, Computer Science), no women applied. There were a few salient differences between natural and social scientists at Hunter: natural scientists appeared to receive larger start-up packages, more research space, and lower teaching loads than social scientists.

The Sponsorship Program was a pilot program. It accepted all applicants who were willing to make the commitments we required because we wanted to help all those who were interested in joining and we wanted to have an impact on the institution. The program included several different components that operated simultaneously. That choice was deliberate – we wanted to maximize our chances of helping the women in the program. The literature also suggests that a program with many components increases the likelihood of including a component that will resonate with someone, even if other components do not. An exploratory analysis of what led to increases in diverse representation in large firms suggested that most diversity was seen in companies that included a variety of mechanisms (Marquis et al., 2008). We understood that our pilot could not isolate which components were necessary or sufficient for success. For example, our workshops included some role-playing, which some attendees found very helpful and others did not. Our aim was to develop the program over time.

### Outcome measures and causal inferences

The main outcome measure was individuals’ research productivity pre- and post-participation in the Sponsorship Program. It is inherently challenging to claim program effects on outcome measures in the absence of random assignment to treatment and control groups. Our research design did not meet these conditions, but approximated a particularly powerful and respected class of quasi-experiments–the regression discontinuity pretest-posttest design–in which causal effects can often be inferred. These are cases in which the treatment is novel, distinctive and abruptly instituted, and the outcomes of interest can be measured directly before and after the intervention begins (Cook and Campbell, 1979). In our view, the Sponsorship Program has these elements. There are some outcomes that, in their temporal proximity to the program, their distinctive character, and their conceptual relation to the program, can plausibly be attributed to the program. For example, the program required certain activities on the part of associates, such as keeping a work log and submitting internal grant proposals. Associates completed all required activities, even though many of those activities were new to them. Simply making an activity obligatory – setting an injunctive norm (Schultz et al., 2007) – was sufficient to change behavior. We recognize that the absence of a control group precludes drawing causal conclusions. We made sustained efforts to construct a matched comparison group *via* curricula vitae (CVs) from other CUNY faculty. At that time, however, CVs were not broadly accessible on websites and only one faculty member responded to our offer of gift certificates. (We believe that this reflected the lack of a professional identity and concerns about underachievement on the part of CUNY faculty.)

## Sponsorship Program: Quantitative and qualitative results

Analyses of quantitative and qualitative data we collected over 7 years—including numbers and kinds of contacts with sponsors; monthly progress reports of paper, proposal, and presentation submissions and outcomes; regular interviews with sponsors; regular collection of updated CVs; outcomes of tenure and promotion processes; periodic survey results and other assessments of associates and their sponsors—suggested that between two-thirds and three-fourths of all associates’ research productivity improved during their time in the program and for some time after their participation in the program ended.

### Research productivity

Data analyses revealed noticeable, broad-based improvements in research productivity. Associates in cohorts 1 through 5 submitted significantly more papers and grants during their first year in the program – Year 1 – than they did during the year before entering the program – Year 0. In Year 2 they submitted significantly more papers and grants than in Year 0. From June 2002 to April 2008, GEP associates became increasingly adept at applying for and obtaining internal funding and were awarded over $4.9M in external grant funding, more than six times what the GEP invested in these associates. During the life of the program, 13 of the 14 eligible GEP associates who came up for tenure were awarded tenure, all nine of those who came up for promotion to associate professor were promoted and two associate professors were promoted to the rank of full professor. (At least three others were promoted to full professor beyond the award period.)

Associates learned to work through procrastination and lack of confidence in order to write, rewrite, and share their drafts of papers and grant proposals. Ultimately, most associates published the major articles or books that had stymied them up to that point, succeeded in obtaining grants to support their research efforts, and were promoted to full professor. Some participants rose to leadership positions in their disciplines and at Hunter College and CUNY; one left Hunter for a position in a more research-intensive institution.

### Effort and achievement

Two correlations reveal the connection between effort and achievement. The number of grant proposals (internal and external combined) submitted in Year 1 was positively correlated with the number of articles accepted for publication in Year 2. Across all years of program participation, the total number of academic articles and grants submitted was positively correlated with the number of internal grants funded, and with the number of journal articles, chapters, and books accepted for publication. Associates had the skills necessary to succeed in publishing their work; effort led to success.

### Sponsor effects

Correlational analyses also indicated the importance of the sponsor. The amount and type of interaction with sponsors was related to subsequent grant submissions and grant getting. For example, across all years of participation in the program there were significant correlations between the number of associate-sponsor phone calls and email exchanges and associate productivity. Those exchanges (but not in-person meetings) were positively correlated with both the number of internal and external grants submitted by associates and the numbers of internal and external grants funded. There was no correlation with the numbers of journal papers submitted or published; the effects of calls and emails appeared to be specific to grant activity.

Face-to-face interactions showed different effects. In-person contacts with sponsors during Year 1 were positively correlated with the number of journal articles, chapters, and books submitted by associates in subsequent years. In several cases, the year-to-year improvement was sharp—from one journal submission to five, for example—strongly suggesting a program effect. Taken together, our data suggest that face-to-face interactions between sponsors and associates were more important for productivity in Year 1, whereas email and phone exchanges became more important in later years, when relationships were better established.

### Collaborations with students

Throughout the Sponsorship Program, we strongly encouraged and tracked collaborating with students at all levels, especially undergraduates, to whom all our associates had access. Many of our associates’ students presented their research at conferences and some became authors or co-authors of published papers. The 30 associates in cohorts 1–5 reported supervising more than 150 undergraduates, 40 MA students, and 25 PhD students during the award period.

### Program evaluations

Associates provided uniformly high evaluations of the Sponsorship Program over the 6-year course of the award. Those high evaluations could be seen as experimenter demand (it would be hard for associates to tell the developers of the program that they were no help) combined with associates’ need to justify the time that they were committing to the program. The positive results already described, however, argue against that interpretation. When rating components of the Sponsorship Program in terms of their usefulness and contribution to the associates’ professional development, associates rated funding for research most highly, followed closely by advice from the GEP directors outside of the workshops, followed by sponsor benefits, workshops, workshop handouts and readings, and interactions with other associates. All of these elements were rated over 4 on 5-point scales where 5 was most effective.

### Associate comments

Unsolicited comments by associates poignantly capture how the GEP helped them navigate rejection of their articles and get their work published; overcome feelings of overwork, isolation, depression, and disconnection from their work; and clarify their professional goals. Examples of comments include reflections on the differences in associates’ working lives as members of the program, such as an increased knowledge and appreciation of what it took to succeed. An unexpected benefit was the sense of community associates told us they developed with fellow participants in their cohorts. For some faculty, the community of GEP associates was the only real community they felt they had at the college. Several participants created professional bonds with their sponsors and thereby expanded their professional networks; even more participants expanded their circle of advisors to include the GEP co-directors and other leaders in the college. Subsequent research suggests the importance of learning communities for women in research-intensive institutions who seek professional legitimacy and advancement (O’Meara et al., 2018).

Even in those Hunter departments with relatively large percentages of women, women felt under-supported (and worse). Some felt that they had no allies, let alone potential collaborators, in their departments, and thought that no one at the college understood, appreciated, or facilitatied their work. Meeting people from different disciplines and departments with similar experiences showed associates that they were not alone in the challenges they faced. In the GEP, women met at least monthly in interactive workshops with peers who were eager to support, connect with, and learn from each other. Recent research shows that faculty learning communities perform important functions, especially for marginalized groups, by creating positive conditions for building academic legitimacy and instilling a sense of belonging (O’Meara et al., 2018).

### Workshops and faculty lacunae

In preparation for a workshop regularly offered in early spring, associates identified and interviewed a scholar in their discipline whose research career they admired, with a focus on how these scholars used summers to advance their work. Associates heard that successful scholars worked regularly on their scholarship for at least two hours every day. They used strategies and techniques to avoid distractions and disrupt procrastination, and enlisted support, including paid help, to ensure that their research time was productive. Almost all faculty at Hunter had degrees from highly ranked, research-intensive institutions, so one might have expected them already to know this, but they did not.

We saw other examples of unexpected gaps in faculty knowledge and skills. At one workshop we conducted for faculty across CUNY, a new faculty member from a different college expressed surprise that receiving a “revise and resubmit” message from a journal was a positive response and that outright acceptances were rare. “You mean I should be happy about that?” she said. One sponsor told us of going over a rejection letter from a journal with an associate, helping her see that she could respond to most of the points without much difficulty, and letting her know that criticism was common and could be handled. We believe that the women faculty in the Sponsorship Program lacked enough such experiences as graduate students or post-docs.

In another workshop, on conference attendance and presenting one’s work in professional settings, we saw first-hand that associates were unaccustomed to talking about their research, even in low-stakes settings. It was stressful for some of them to present succinct synopses of their work or craft engaging introductions to their conference presentations. Some associates talked about the embarrassment of attending conferences and seeing researchers with whom they had attended graduate school, researchers who were now far ahead of them in their research accomplishments. They had begun to wonder if they were capable of conducting major research projects. Had they been capable, the world seemed to be telling them, they would have been hired at a research-intensive institution to begin with. Our message was that, with strategy and planning, they could make attending conferences advance their work, their visibility, and their careers.

### Lessons Learned: 1–3

#### Lesson learned #1

*By focusing on skill development rather than talent, and by providing necessary information*, the Sponsorship Program provided a different message than the one our associates had internalized during their professional socialization. Its message was that success in academia is the result of learning what to do – and there is a lot to learn! – and setting aside time to practice doing it. The Sponsorship Program, *via* its interactive workshops, assignments, and readings, was explicit in dissecting the skills and information necessary for professional success in academia.

With its focus on skills and information the Sponsorship Program sidestepped issues of talent. Its message was that one could learn how to develop one’s ideas and present them effectively; one could learn how to be a good leader; one could learn how to respond to rejection. Similarly, the Sponsorship Program fostered the idea throughout the college that participating in faculty development programs/learning communities added value to a faculty member. Associates listed Sponsorship Program membership on their CVs with pride. Over time, candidates’ participation in the GEP was increasingly framed as an asset by their department chairs in their presentation for tenure and promotion in college-wide proceedings. As the Sponsorship Program demonstrated its effectiveness and gained prestige, chairs used it as a selling point in recruiting new faculty.

#### Lesson learned #2

*Faculty benefit from a circle of advisors* – people from different backgrounds who have different perspectives, skills, and knowledge – rather than a single mentor. The use of expert sponsors who were compensated fairly for their efforts encouraged sponsors to commit time and effort to their mentoring and encouraged participants to ask for help when they needed it, especially in grant preparation and paper submission. The use of expert sponsors in the associates’ specific intellectual and professional areas addressed some of the challenges associates faced due to professional isolation and a dearth of natural colleagues at Hunter.

But a single sponsor is not enough. A serendipitous feature of Hunter’s GEP—that the three co-directors differed in their knowledge, experience, and interpersonal styles and played different roles in dealings with associates, senior administrators, and ADVANCE—turned out to be a crucial ingredient in how we mentored individual associates, what and how much we were able to do to help them, and how much they took from the program. Given the important role that the associates played in each other’s development, we increasingly appreciated the benefits of peers with whom one can check in regularly, at an appointed time, to discuss work progress or work problems, exchange drafts of work, or get advice about career moves.

#### Lesson learned #3

*Sustained connection to professional networks is necessary for career success*. The GEP did not recognize the importance of this early enough. Without regular interactions with people with common professional backgrounds, understandings, interests, and concerns, it is easy for scholars to feel isolated and fall behind. Simply keeping up with the literature in one’s field has become increasingly daunting as papers proliferate; a network in which people mention useful articles to each other fosters being tuned in. One possible benefit of the ongoing COVID-19 pandemic is increased creativity with respect to conferences and meetings. The increased normativity of long-distance connections could be profitably used to create networks for scholars.

## Sponsorship Program: Whose research programs benefited, whose did not, and why

### Large gains in research productivity

We define large gains as discontinuous jumps in levels of research activity that resulted in scholarly products (grants, articles, books). We classify our program as a success because roughly two-thirds to three-quarters of the associates showed large productivity gains during the measurement period (2002–2008) and many continued those gains, continuing to publish their research and apply successfully for funding. All associates took their participation seriously and filed monthly progress reports, and most honored the commitments that came with Sponsorship Program membership. From subsequent informal interactions with associates, we have come to think that the people who were helped by the Sponsorship Program were greatly helped. No doubt they were ready to be helped, or they would not have applied to the program, and their sponsors were a good match, but they continue to express informally to us the idea that their sponsors’ and our support of and belief in them, and the confidence this inspired in them, helped them succeed.

### Medium gains in research productivity

About a quarter to one third of the associates showed medium gains. These associates increased their research engagement and scholarly work but their work products were limited in nature and scope and did not change the trajectory of their research programs or professional careers. Although modest gains may be expected from any intervention program, we cite two possible reasons for limited results. One was that the match between the sponsor and the associate was not always ideal. Some sponsors turned out to be ill-suited to an associate’s current research topic or to the methods, techniques, or analytic strategies that the associate needed to learn. In other cases, the pair did not interact as frequently as they might have, usually due to associate shyness. (The mean number of contacts per month for all associates in the first 2 years of sponsorship was 4.2.) Over the past 20 years, some associates changed their activities to align better with values that were more important to them than producing scholarship; some have become department chairs and program heads or have taken other leadership roles in the college or in their fields. They have not abandoned research but they are classified as having made medium gains because our measuring rod only measured one thing – research productivity as it is traditionally defined.

### No gains in research productivity

Three individuals showed no measurable research productivity gains, even after more than one year in the program. Two associates discovered that they had not fully realized what was required to be a productive researcher. They had formerly attributed their lack of research productivity to lack of time and support, but discovered that their values lay elsewhere. Those two redirected their efforts to teaching, mentoring, leadership, and service. For them the benefit of the Sponsorship Program was to clarify and readjust their professional goals. A third associate who joined the Sponsorship Program in Year 5, the year she was coming up for tenure, withdrew her candidacy and subsequently left the college and academia.

### Lessons Learned: 4–7

#### Lesson learned #4

*The traditional scholarly norms in the sciences do not fit everyone*. We used classic productivity metrics as our measures of success because those metrics were highly respected by both the faculty and the leadership of the college. Although we appreciated work highlighting not only the scholarship of discovery but also the scholarships of integration, application, and teaching (Boyer, 1990), championing such forms of scholarship seemed outside NSF goals. We thus did not emphasize their potential value nor did we integrate those forms into the Sponsorship Program. For some associates other forms of scholarship were likely a better fit.

#### Lesson learned #5

*Succeeding in one’s discipline and succeeding at one’s institution are not the same thing*, especially in predominantly undergraduate institutions. For women (and men) to excel in their research careers, faculty development programs need to encourage and support the use of disciplinary, as well as institutional, standards, practices, and expectations. Success along the tenure track requires a mix of strategies, advisors, resources, and other supports, depending on what counts as success in one’s field and one’s institution. At Hunter, as in many other teaching-intensive institutions, research, teaching, and service to the college, the department, and the discipline all count toward tenure (though not necessarily toward promotion) decisions. At Hunter, promotion to associate professor and especially to full professor rests largely on research productivity. Informally, being perceived as a good citizen and good colleague factors into tenure decisions in some departments. It was important for our associates to learn college and departmental norms and develop efficient and effective ways of meeting those standards while also meeting the professional standards of their disciplines if they wanted to achieve full professorship.

#### Lesson learned #6

*Academia needs broader models of career success than those that are dominant in research-intensive institutions and national funding agencies.* Teaching-intensive institutions are not failed research-intensive institutions. They are fundamentally different in their missions, values, structures, and resources. Increasing research support and scholarly activity among women and BIPoC faculty at teaching-intensive institutions will enable these institutions to remain vibrant by attracting and retaining strong faculty, creating opportunities for collaborations with undergraduate and master’s students, and inspiring students to aim higher. Teaching-intensive institutions can assert their own norms and standards of academic excellence by explicitly broadening the range of high-quality scholarship and creative activity that is supported and rewarded to include scholarships of integration, application, and teaching, among others. Seattle University, a teaching-intensive institution, used its 2016 ADVANCE award to better align its expectations of faculty and its promotion standards with its educational mission and successfully achieved that goal in 2021 https://www.seattleu.edu/advance/.

*Longer-term effects*. About half of the sponsored faculty continued to make strides in the short term and in the long term. For the other half, however, the benefits of the GEP lessened over time as demands of the college became more constraining, both because of increased expectations for faculty as they move up the ranks and because ambitious teaching-intensive and research-oriented colleges like Hunter try to do it all, and have high expectations for teaching, research, and service. It was difficult for former participants to maintain their research. The scheduled workshops and GEP directors were no longer actively available. Lack of bridge funding was another obstacle to continued research activity. If a faculty member in the social and natural sciences lost external grant funding, they then returned to teaching three courses a semester, at which point it became difficult to perform the pilot research necessary for obtaining future funding. It was even more difficult to maintain the sense of community that the Sponsorship Program provided, a difficulty exacerbated by teaching at a commuter campus.

Even obviously successful programs cannot continue at institutions that do not have the funds to maintain them. Mentoring, sponsoring, and supporting faculty cost time and therefore money. Supporting research, including research with students, costs money. At teaching-intensive institutions, the needs are so strong and so pressing that supporting faculty and research seems like an unaffordable luxury. When NSF support ended, funds were not available at Hunter to maintain the staffing, the release time for associates, the modest research funds, the money for sponsors, or the workshops.

#### Lesson learned #7

*Women and people of color need ongoing opportunities for intellectual and social community outside of formal academic department structures.* The salutary effects of even demonstrably successful programs may not endure once the program has ended. Academic departments are not optimally designed to offer support and a sense of belonging for people from underrepresented groups. The historic disadvantages, inequities, and biases that women and people of color face in academia do not disappear at the end of a program. The idea that a single, even multi-year, intervention can forever redirect, support, and sustain a successful academic career in the face of accumulated disadvantage is not tenable.

On analogy with efforts to deal with the COVID-19 pandemic, we propose that people need regular “booster shots” throughout their careers to maintain forward momentum in environments not built for them—such as learning communities, workshops, retreats, circles of advisors, and other regularly occurring opportunities.

## Effects of the Gender Equity Project on academic departments, the College, the University, and beyond

### Department chairs and departments

Academic departments are where faculty live their professional lives, and departmental conditions generally and the department chair particularly have an outsized effect on faculty productivity and satisfaction. During the time of our award, departments and department chairs at Hunter College differed considerably in their support for research activity and their focus on gender equity and faculty satisfaction.

Department chairs at Hunter College are elected, serve for renewable 3-year terms at the pleasure of the voting members of their departments, have more responsibilities than authority, and are under-compensated for the nature and scope of their work. As a former department chair and provost at Hunter College, one of us (VCR) can speak authoritatively about the position of chair.

The responsibilities of department chairs are: to provide a schedule of classes that meets student needs and college requirements; to staff courses with strong teachers; to supervise the department staff; to make committee assignments and ensure that committees do their work; and to arrange for the regular evaluations of faculty and staff. In 2001, chairs received no incentives (or even encouragement) to create faculty development opportunities, increase the time faculty spend on research, or nominate faculty for awards.

Our experience in the GEP was a window into how academic departments function to shape careers. Hunter’s faculty, female and male alike, are committed to Hunter’s mission. They are dedicated teachers and mentors. Hunter attracts faculty who want to make and do make a difference in students’ lives. Department chairs and deans rely on their faculty’s willingness to put their students first.

In 2001, chairs varied in their support for faculty research. Some chairs saw faculty research as at odds with the core mission of the college, and, therefore, the core mission of their department. Some department chairs were – quite reasonably – concerned about the costs of losing the associate’s teaching due to course release. Some chairs were thus concerned that the GEP would expose associates to new norms, for example, about teaching workloads or other conditions of work. Knowing of other norms could create resentments among their faculty. Other chairs may have been concerned about the exposure of potentially negative aspects of their leadership or their departments to outsiders.

One faculty member remarked that her new chair changed her conception of the role of chair. Up to that point she had simply been happy when a chair did not put obstacles in her way. Neutrality was the most she had hoped for from a chair. The new chair arranged for her to be nominated for fellow status in a professional society. The idea that a chair would care about professional opportunities for her, as her new chair did, was shocking. Unsupportive chairs exist at all types of institutions and are not necessarily more frequent at teaching-intensive institutions. But their effect is amplified at teaching-intensive institutions.

Some chairs saw their departments as already equitable; they did not see a problem that needed to be fixed. Those chairs were openly skeptical about our comprehensive Sponsorship Program. Knowing from psychology that people are more likely to behave as allies if they are treated as such (Brickman, 1987), our approach was to treat chairs as allies or partners in supporting their faculty. More crucially, we strongly advised our associates to treat their chairs (and other members of their departments who might become chairs or become members of committees that would affect their futures!) as partners and allies, regardless of how they currently felt about their level of support, while being aware of the fact that chairs or senior faculty might rate their needs as unimportant compared to the department’s. In a workshop on negotiation, we introduced the idea that associates who wanted something from their department could show how that could lead to solving a departmental problem.

There were times when either Rabinowitz or Valian intervened in what appeared to be discriminatory or hostile conditions, for example, in a department in which several associates complained about a male staff member’s sexist behavior. We were generally helpful in resolving such issues. Over time, we were able to make the case that the GEP was not a threat to department chairs or departments. When departments learned of our efforts to rationalize certain college procedures and make them more transparent and to improve orientation for new chairs, they saw benefits of the GEP. At one departmental presentation that we gave, a faculty member said, “you’re like an ombuds for departments,” a compliment we highly valued.

### Lessons Learned: 8–9

#### Lesson learned #8

*Gender equity and diversity programs are windows into institutional effectiveness*. A focus on the perceptions, conditions, and outcomes of White women and BIPoC faculty reveals an institution’s strengths and vulnerabilities. With that focus, the GEP could see how departments and offices did and did not function for women faculty and for *all* faculty. In the case of the GEP and Hunter College, the focus on gender equity revealed that, despite the nearly equal representation of women and men, women languished in the ranks of associate professor in many departments, were less satisfied and felt less supported than men, and were less productive as scholars. Inadequate and inconsistent information and support for all faculty, starting with offer letters and continuing through tenure and promotion proceedings, had a disproportionate impact on women’s careers.

#### Lesson learned #9

*There are advantages and disadvantages to running a major program outside of the formal organizational chart*. The imprimatur of the National Science Foundation and the nature and size ($3.75M) of the award conferred prestige on the GEP and its co-directors, and multiplied its effects on the institution and individuals. Investments by NSF (and Hunter) were evident in the time commitment of the co-directors and the refurbished, dedicated program space that offered participants privacy, safety, and social support. All the elements that made the Program seem special also boosted morale and confidence and were ultimately important to its success.

Operating outside of the formal organizational chart, with no direct reports within the college, gave the co-directors autonomy and standing throughout the college. Faculty trusted us with information (about people, policies, and practices) that would otherwise not have been formally reported, and they trusted us to act in the interests of people within and beyond the Sponsorship Program. We had standing to intervene, within limits, as ombudspeople, and we had access, within limits, to the Hunter and CUNY leadership. The significant disadvantage was our inability to institutionalize GEP initiatives, whether they were resource-intensive programs like the Sponsorship Program or relatively inexpensive activities like data collection and reporting after the award period. Nor could we raise money independently of the college.

### Hunter College

Our goal—and NSF’s mandate—was to transform an institution. *Via* the Sponsorship Program, the GEP directly served 30 associates. The GEP’s larger efforts touched hundreds of people and altered numerous policies and practices, not just at Hunter College but across the 25 units of the CUNY.

The GEP was committed to transforming institutional policies and practices in order to create uniform and rational expectations, and knowledge of those expectations, for all faculty. We expected those changes to improve conditions for research at Hunter College and CUNY, and we worked to sustain those changes. What we learned from the Sponsorship Program, from being part of ADVANCE, and the growing literatures on gender equity, racial disparities, and advancement in higher education all contributed to this effort. We summarize below the major changes in policies, practices, and programs that were launched by the GEP during the course of the ADVANCE award, many of which persist in some form to this day.

• We instituted gender equity benchmarks at Hunter College. Throughout the award, the college collected and reported data on hires, advancement, and faculty flux by gender in the relevant natural and social science departments in the School of Arts and Sciences.
• After analyzing offer letter to scientists at Hunter College, the GEP discovered wide disparities from department to department in how much relevant information was included in any given offer. To ensure uniform and complete offer letters, the GEP created a checklist of items an offer letter should include and sample narrative templates for the school deans to provide to all chairs. As Hunter Provost, with the support of the college president, Jennifer Raab, Rabinowitz instituted the practice that all offer letters to new professorial-rank faculty members at Hunter College must include start-up funds for research in order to establish a research expectation.
• Working with the Provost’s Office, the GEP developed Tenure and Promotion Guidelines for the College (taken from documents in the Provost’s Office, the Hunter Faculty Handbook, and a review of exemplary tenure and promotion packets), a new Chair Handbook, and guidance about pathways to success in various disciplines.
• The GEP, working with chairs and the Provost’s Office, developed a survey, known as the “Progress and Planning Report,” that natural and social science departments used to report their efforts toward equity and diversity on an annual basis. All science chairs agreed to provide the data with the understanding that the administration would use the information as one criterion in assigning faculty lines and space. For what was perhaps the first time, chairs now knew what the administration expected of them in advocating for lines in their departments. Other items on the report included lists of all faculty whom the department had nominated for awards, honors, or memberships in prestigious organizations, and all faculty who received such accolades. Departments also listed departmental supports that they provided for their faculty. These categories were intended as much to be interventions as reports—to sensitize chairs and their executive committees to best practices in higher education.
• The GEP developed procedures that linked positive efforts toward equity in the Progress and Planning Reports with small cash awards to departments that provided evidence of progress. The money was used for mentoring, colloquia, and so on. It is difficult for faculty who work at research-intensive institutions to understand the meager financial support that departments and programs at underfunded institutions receive. To give an example from 2021, training areas in Psychology at the CUNY Graduate Center were allotted $300 to spend on supplies that would benefit student research.
• The GEP created websites to include equity data, newsletters, resources, and web-based tutorials. Both the GEP website and tutorials have been regularly updated and are currently undergoing reconstruction.
• An outgrowth of the GEP was the creation, in 2007, of a Professional Development Office (PDO) in the Office of the Provost. The PDO institutionalized many GEP initiatives and organized college-wide faculty development and faculty diversity efforts. This included the establishment of a new, permanent administrative line, funded by the college in 2007 and continuing to this day, in the Provost’s Office.
• In 2007, Rabinowitz and Nicols-Grinenko instituted regular workshops on preparing for tenure and promotion that were open to male and female faculty throughout Hunter College. The workshops were always over-subscribed, and had to be offered twice per year because of demand. Some participants attended the workshop more than once to reinforce certain lessons. Following the GEP’s emphasis on skills and information, participants learned how to organize and present a CV in the best style of their discipline, how to write personal statements about their research, teaching, and service accomplishments that would present them to best advantage, and how to work with their department chairs to make their best case. One of the most valuable aspects of the program was sharing models of the tenure and promotion packets of exemplary faculty who had recently succeeded in the process. Over time we developed a library of such materials to satisfy the demand among the faculty throughout the college. As a result of the availability of these models, tenure and promotion packets improved markedly in quality, becoming more comprehensive, organized, and compelling.
• Starting in 2010 the GEP established an annual five-hour New Faculty Orientation at Hunter College for all new professorial-rank faculty. The orientation prepared faculty for tenure and promotion from day one by discussing, among other topics, balancing the roles of research, teaching, and service; time management; and teaching effectively and efficiently.

Some changes occurred through discussion with department chairs. Other changes occurred through more general effects, in which associates became seeds of change in the college. Some former associates of the Sponsorship Program later served as workshop leaders. Some became department chairs who developed procedures that were helpful to faculty. Others shared what they learned about professional development from the GEP informally with colleagues and students.

### Lessons Learned: 10–11

#### Lesson learned #10

*Universal design*, a concept borrowed from architecture, is generally applied to serving individuals with disabilities, but it has more general application. The benefits of making life better for White female and BIPoC faculty end up also making life better for everyone. GEP efforts to help women—institute templates for offer letters, clarify and publicize tenure and promotion guidelines, institute regular reporting on scholarship, provide training for giving presentations and grant-writing, encourage the creation of circles of advisors—helped men as well. Efforts on behalf of scientists helped non-scientists. What worked in the School of Arts and Sciences also worked in the Schools of Education, Social Work, and Nursing and Health Sciences. Universal design, with its broad reach, has the additional advantage of creating—and sustaining—buy-in from most constituencies. Among the legacies of GEP initiatives are policies and practices like standardized offer letters, regular faculty satisfaction surveys, and regular tenure and promotion and manuscript and grant-writing workshops. In these cases, the changes cost relatively little and have universal design features that benefit everyone.

#### Lesson learned #11

Presidents, provosts, and deans play an important role in promoting the linked goals of equity and support for faculty research. Hunter’s new president, Jennifer Raab, was announced just weeks before Hunter’s ADVANCE proposal was submitted in 2001; she wrote a letter strongly supporting its goals. As president, Raab invested in the GEP’s future by completely renovating the space that would become its permanent home. Later, and more crucially, she accepted the recommendation of then-provost Rabinowitz to institute a policy that all incoming professorial-rank faculty would be awarded research funds, however modest, as part of start-up packages, and that start-up funds would appear on the checklist of items to be included in all offer letters to professorial-rank faculty. Standardized offer letters that were co-signed by relevant officers of the college ensured that such funds were guaranteed, ensured that such funds were guaranteed. President Raab and then-Provost Richard Pizer both supported the goals of the award and respected the GEP’s autonomy and responsibilities to NSF.

Faculty development, a crucial piece of equity, can nevertheless be a tough sell in institutions that are challenged to provide quality educations to underserved students. For non-elite, under-resourced institutions of higher education, there is never enough money for everything that’s important. Since priorities change when leadership changes, it is important to develop an understanding of the importance of faculty research for student development and solidify a commitment and capacity to support a diverse, engaged, research-active faculty.

Table 3 summarizes our lessons learned.

**TABLE 3:** Lessons learned.

***Lesson learned #1***
*By focusing on skill development rather than talent, and by providing necessary information*, the Sponsorship Program provided a different message than the one our associates had internalized during their professional socialization.
***Lesson learned #2***
*Faculty benefit from a circle of advisors* rather than a single mentor – people from different backgrounds who have different perspectives, skills, and knowledge.
***Lesson learned #3***
*Sustained connection to professional networks is necessary for career success*.
***Lesson learned #4***
*The traditional scholarly norms in the sciences do not fit everyone*.
***Lesson learned #5***
*Succeeding in one’s discipline and succeeding at one’s institution are not the same thing*, especially in predominantly undergraduate institutions.
***Lesson learned #6***
*Academia needs broader models of career success than those that are dominant in research-intensive institutions and national funding agencies.*
***Lesson learned #7***
*Women and people of color need ongoing opportunities for intellectual and social community outside of formal academic department structures.*
***Lesson learned #8***
*Gender equity and diversity programs are windows into institutional effectiveness*.
***Lesson learned #9***
*There are advantages and disadvantages to running a major program outside of the formal organizational chart*.
***Lesson learned #10***
*Universal design*, a concept borrowed from architecture, is generally applied to serving individuals with disabilities, but it has more general application. The benefits of making life better for White female and BIPoC faculty end up also making life better for everyone.
***Lesson learned #11***
*Presidents, provosts, and deans play an important role in promoting the linked goals of equity and support for faculty research.*

### The City University of New York and beyond

The influence of the GEP spread beyond Hunter College. A team from another CUNY college attended our workshops, developed their own workshops, and later successfully applied for an ADVANCE award. The GEP consulted with a CUNY comprehensive technical college in their successful bid for an ADVANCE award. The GEP applied for and received a Partnerships for Adaptation, Implementation, and Dissemination (PAID) award to extend its workshops to faculty across CUNY, and to develop new grant-writing workshops. It ran those workshops for 3 years. The university-wide response to the workshops over the duration of the program was extremely positive, with mean evaluations of their usefulness 3.55 on a four-point scale. Through a partnership with the New York Academy of Sciences, the GEP ran workshops that attracted over 100 graduate students, post-docs, and junior faculty in the tri-state area; those workshops received very high evaluations as well, and were again very well-reviewed.

When Rabinowitz became CUNY’s University Provost, she established the Office of Faculty Affairs within the Office the Provost, created the new position of University Associate Dean for Faculty Affairs—the first position within Academic Affairs to be focused on the faculty in CUNY history—and hired Nicols-Grinenko to head that office. (Rabinowitz and Nicols-Grinenko strongly supported then-CUNY Chancellor James B. Milliken, CUNY’s then-Chancellor, in his leadership of the historic, successful campaign to lower the university teaching workload of CUNY faculty in 2017.) Nicols-Grinenko has recently become University Dean for Faculty Affairs, signaling a long-term commitment by CUNY to its faculty. There are now regularly scheduled well-attended workshops series and course releases for mid-career faculty seeking promotion to full professor, an institutionalized program to support research among community college faculty, an award program to support faculty who write books, and all-day grant-writing workshops to help faculty sharpen their specific aims.

Measures of faculty satisfaction—followed by action on the part of colleges—have now become routine at CUNY. Rabinowitz, as CUNY Provost, and Nicols-Grinenko partnered with Harvard’s Graduate School of Education’s COACHE program to institute regular surveys of CUNY faculty’s satisfaction, which continue to this day, and CUNY pilot-tested the first major survey of community college faculty satisfaction—now a COACHE staple. With COACHE partners, Nicols-Grinenko and Rabinowitz won a grant from the Harvard Club of New York Foundation to support CUNY faculty participation in Harvard’s higher education leadership programs. Seventeen aspiring CUNY leaders, most of them faculty and administrators of color, attended the 2-week leadership and management programs over a 2-year period without any cost to them. Many participants described the experience as career-changing and went on to major promotions, including to college president. In addition to heading the Office for Faculty Affairs, Nicols-Grinenko now co-directs The Leadership Institute at CUNY (TLIC), a Mellon Foundation-funded program that supports faculty at CUNY and across the nation who wish to become leaders in urban, mostly teaching-intensive, higher education institutions.

## Implications for the future of programs to advance gender equity

As we have noted throughout this paper, the challenges for women and people of color at teaching-intensive institutions overlap but are also distinct from their counterparts at research-intensive ones. Increasing representation remains important, especially in some departments. Even more important is increasing faculty’s ability to develop their scholarship and engage students in meaningful inquiry.

### Facilitate cross-institutional and collaborative work

Teams and cross-institutional collaborations are growing in size, averaging more than three people in some scientific fields, and becoming more cross-institutional (Jones et al., 2008; Stewart and Valian, 2018). Yet female faculty in teaching-intensive institutions are unlikely to be part of cross-institutional teams. Scientists from top-tier institutions tend to collaborate with scientists from other top-tier institutions—and the same is true of scientists in lower-tier institutions in what Stewart and Valian call “assortative matching.” We can also see this as contributing to the accumulation of advantage and disadvantage, respectively. The 5% of institutions in the top tier of citation rates accounts for 59% of cross-institutional collaborations; the lowest tier, which consists of 80% of all institutions and virtually all teaching-intensive institutions, accounts for just 30% of cross-institutional collaborations (Jones et al., 2008).

Women are, on average, less likely to adopt the collaborative patterns—maintaining regular and repetitive collaborations over time, finding new collaborators to plug structural holes in their knowledge base as needed—that are related to success (Jadidi et al., 2018). Given the scientific imperative to develop and maintain stable, trusted collaborative networks (Carr and Walton, 2014; McDaniel and Salas, 2018)—and the already existing marginalization and isolation of female and minority scientists in teaching-intensive institutions—it is unlikely that useful collaborations involving faculty and students from teaching-intensive institutions will take place without concerted and deliberate action on the part of major funding agencies like NSF. To that end, ADVANCE has supported four partnership grants over the past 20 years, three within STEM disciplinary professional societies and one among different types of institutions.

Cross-institutional collaborations that seem particularly promising include those in which there are strong and durable ties among colleges, as in public systems with flagship research units and regional, satellite, and community colleges. Others with potential include research-intensive institutions that already have some relationship with teaching-intensive institutions by virtue of geographic proximity or other important commonalities, like shared interests in regional or global challenges.

For collaborations to work, recognition and respect for the talents and skills of all partners in the collaboration are required. Work on diverse teams shows that the innovative solutions that diverse teams generate occur when participants have a feeling of psychological safety and a feeling that what they have to offer is valued (see discussion in Stewart and Valian, 2018, Chapter 2). People do not offer ideas unless they think that those ideas will be respectfully considered. In order to receive funding, potential teams would describe how they intend to maximize productive collaborations. At the faculty level, the benefits are obvious for both types of institutions. At the student level, the benefits include giving undergraduates at teaching-intensive institutions familiarity with the doctoral institutions where they might apply for graduate school and giving those research-intensive institution access to a wider and more diverse range of students than generally apply.

### Include women in the social sciences

The ADVANCE program started 20 years ago in response to obvious and serious problems in the sciences, primarily the natural sciences, engineering, technology, and mathematics: women were underrepresented, underpaid, under-tenured, and underpromoted. The early leadership of ADVANCE was mindful of the relevance of social science research to the success of the program, and the program included all NSF-supported disciplines, including the social sciences, from its inception. Our experience suggests that the plight of women in the social sciences remains underappreciated generally, much like the plight of women in teaching-intensive institutions—and for much the same reasons: that women are more plentiful in the social than the natural sciences and seem to be doing “well enough.” We suspect that the social sciences are also regarded as less rigorous and important than the natural sciences, and for all these reasons, less in need of attention and interventions.

Two recent articles on the plight of women in the social and behavioral sciences document that women continue to experience significant gender inequities despite their strong representation in these fields (Casad et al., 2022; van Veelen and Derks, 2022). Data on citations, awards, promotions, salary, and invitations to give colloquia and keynotes suggest that women in the social sciences are under-recognized (Beaulieu et al., 2017; Nittrouer et al., 2018; Ginther and Kahn, 2021; Gruber et al., 2021; Skitka et al., 2021; White et al., 2021). In the same way that we have spoken of institutions like Hunter College as places where the hidden problems predominate over overt problems, we see the social sciences as disciplines where the hidden problems predominate. The situation of women in the social sciences is the leading edge. Social science is important to institutions because of what social science can uniquely contribute to our understanding of individual and institutional change. But it is also important because the social sciences represent such a large percentage of faculty and students in most colleges, and they continue to attract a large percentage of undergraduate women. Women in social science face challenges that all women face in professional life. We thus recommend expanding the focus of attention to include *hidden problems*, by increasing funding opportunities for teaching-intensive institutions and for women in the social sciences.

### Final thoughts: transforming institutions; potential roles for funding agencies

NSF’s ADVANCE Institutional Transformation program has increased the numbers of women in STEM. The 2001 and 2003 cohorts, for example, increased the percentage of women from 16% to 24% and of new women hires from 25% to 35%. Comparable increases from comparison groups of non-ADVANCE institutions were significantly lower (Rosser et al., 2019).

As crucial as it is to increase the representation of women in science, increased representation is but one facet of diversity, equity, and inclusion. Similarly, as important as it is to support the research productivity of women and people of color in science, discrete efforts like the Sponsorship Program, no matter how well-intentioned and well-designed, cannot alone create real and lasting change in the research careers of minoritized groups. As is increasingly being realized, our notion of transformation itself needs to expand to encompass strategies for creating and sustaining comprehensive, inclusive work environments for all kinds of people over long periods of time. If our experience is a guide, we believe that teaching-intensive institutions will be particularly challenged to sustain improvements in research environments beyond the 5-year award period that NSF IT awards provide. The GEP’s Sponsorship Program was successful in improving scholarly productivity, but no amount of faculty development can overcome inadequate facilities, underfunded research operations, poor incentive structures, and the lack of intellectual community and research collaborations that sustain successful academic careers. Five or even 10 years is not enough time to transform the institution in the ways we now understand that institutions must change. At colleges like Hunter, where the pressures from the institution are unremitting and where the deck is stacked against research productivity from the get-go, faculty need a constant prevailing counter-force in order to be successful in the external world.

All institutions have four potential sources of funding: grants from federal and other agencies, state and city support, private philanthropy, and tuition. Public teaching-intensive institutions like CUNY colleges are, by their nature, dependent on tuition, and that tuition—now about $7,500 per year for full time attendance at CUNY–does not cover the cost of instruction, let alone undergraduate research opportunities, faculty research, or faculty development. The infrastructure to support expensive science is lacking and state and local governments are increasingly chary with funding. To improve equity at those institutions, and to ensure a future in STEM for the diverse group of immigrant students, first-generation students, and students of color, more extended support is needed.

One way federal and state funding agencies can evaluate applications for continued support from teaching-intensive institutions is to include the economic and social mobility of its students as a criterion. As we have noted, higher education is the best engine of mobility. To maintain and enlarge the range and effectiveness of the teaching-intensive institutions that provide that mobility, more funding for the researchers at those institutions is warranted and necessary. Federal agencies already have some mechanisms. We think they can be expanded.

We make two further recommendations. Funding agencies understandably focus on new ideas, and thus do not engage in long-term funding of successful faculty development programs at teaching-intensive institutions. One way to prevent the return to *status quo ante* is to continue support at under-resourced institutions that can demonstrate the effectiveness of their programs. The second recommendation is to require institutions to include a commitment within the initial application to seek outside funding, if necessary, to sustain demonstrably successful faculty development projects. In that way, effective programs could be immune to changes in administrative leadership priorities.

In the ecosystem of teaching-intensive institutions, student success affects faculty and faculty success affects students. Reflecting on the meaning of faculty research success to students, one of our former associates, now a full professor, recently wrote:

“*Students at Hunter are also extremely proud of having been a student of mine or others that are published, in the news, or have a presence in policy circles. Comments go something like this: “I go to Hunter and I get to take classes with famous professors, too”. Or: “I see myself in your research.” Faculty development should be a higher priority for Hunter because it inspires students. I wonder if anyone has ever surveyed students on their feelings about faculty.”*

We are not aware of such studies, which go far beyond regular teaching evaluations, but we offer this as another fruitful area for future research.

## Author contributions

All authors listed have made a substantial, direct, and intellectual contribution to the work, and approved it for publication.

## Footnotes

1 The Carnegie Classification of Institutions of Higher Education includes 32 different types of not-for-profit schools. Three types – a total of 469 institutions – confer advanced degrees: R1 schools (*n* = 146) that engage in very high research activity top the list, followed by R2 schools (*n* = 134) that engage in high research activity, followed by another category that includes schools that confer doctoral or professional degrees in a small number of fields (*n* = 189). These 469 institutions enroll about 41% of all United States students at all levels from associate’s to doctoral sectors and confer over one third of all bachelor’s degrees (Lombardi and Craig, 2017; Carnegie Classifications, 2021), a total of 7,817,409 students (calculated from Carnegie open data, https://carnegieclassifications.iu.edu/downloads.php). The remaining 29 types of schools educate everyone else, a total of 11,474,598 students enrolled in 3,471 schools (similarly calculated).

2 Hunter College was founded in 1870 as a women’s teaching college. As detailed in Valian (2020), Hunter was a hospitable environment for intellectually ambitious young women. Hunter educated two Nobel Prize winners (Gertrude Elion and Rosalyn Yalow) - the only school with that distinction - and many other important scientists (including Mildred Spiewak Dresselhaus, Beatrice Mintz, and Mina Spiegel Rees). Between 1900 and 1940, Hunter graduated 8% of the women who went on to get a Ph.D. in mathematics. Hunter is now one of 25 colleges and schools that comprise the City University of New York, the nation’s largest urban public university, and arguably the most diverse university in the world. In 2020, Hunter’s undergraduate student enrollment was nearly 18,000 students, of whom 65% were women and 70% were students of color (http://www.hunter.cuny.edu/communications-office/Hunter_Factbook_2020.html). As in many other predominantly undergraduate institutions, Hunter’s professoriate has a relatively large percentage of female full-time faculty (now over half, or 54%) and almost one third (31%) are faculty of color (http://www.hunter.cuny.edu/communications-office/Hunter_Factbook_2020.html). At the start of the NSF award period, slightly less than one half of Hunter’s full-time faculty was female.
